# *Plasmodium knowlesi* Malaria in Humans and Macaques, Thailand

**DOI:** 10.3201/eid1710.110349

**Published:** 2011-10

**Authors:** Somchai Jongwutiwes, Pattakorn Buppan, Rattiporn Kosuvin, Sunee Seethamchai, Urassaya Pattanawong, Jeeraphat Sirichaisinthop, Chaturong Putaporntip

**Affiliations:** Author affiliations: Chulalongkorn University, Bangkok, Thailand (S. Jongwutiwes, P. Buppan, R. Kosuvin, U. Pattanawong, C. Putaporntip);; Naresuan University, Phitsanulok, Thailand (S. Seethamchai);; Vector Borne Disease Training Center, Saraburi, Thailand (J. Sirichaisinthop)

**Keywords:** malaria, epidemiology, Plasmodium knowlesi, parasites, mixed-species malaria infection, Thailand, merozoite surface protein 1, small subunit ribosomal RNA, cross-transmission, humans, macaques, research

## Abstract

Naturally acquired human infections with *Plasmodium knowlesi* are endemic to Southeast Asia. To determine the prevalence of *P. knowlesi* malaria in malaria-endemic areas of Thailand, we analyzed genetic characteristics of *P. knowlesi* circulating among naturally infected macaques and humans. This study in 2008–2009 and retrospective analysis of malaria species in human blood samples obtained in 1996 from 1 of these areas showed that *P. knowlesi* accounted for 0.67% and 0.48% of human malaria cases, respectively, indicating that this simian parasite is not a newly emergent human pathogen in Thailand. Sequence analysis of the complete merozoite surface protein 1 gene of *P. knowlesi* from 10 human and 5 macaque blood samples showed considerable genetic diversity among isolates. The sequence from 1 patient was identical with that from a pig-tailed macaque living in the same locality, suggesting cross-transmission of *P. knowlesi* from naturally infected macaques to humans.

## Abstract 2

This parasite may be transmitted from macaques to humans.

*Plasmodium knowlesi* circulates mainly among long-tailed macaques (*Macaca fascicularis*) and pig-tailed macaques (*M. nemestrina*) that inhabit a wide area of Southeast Asia (*1*). Microscopy-based detection of *P. knowlesi* has failed because morphologic features of young trophozoites of *P. knowlesi* resemble those of *P. falciparum* and characteristic band-shaped growing trophozoites resemble those of *P. malariae* (*2**–**4*). To date, the effective tool for diagnosing *P. knowlesi* infection is PCR specific for multicopy genes, such as small subunit rRNA and mitochondrial cytochrome *b* (*3**–**5*).

Human infections with *P. knowlesi* vary by geographic location (highest prevalence in Malaysian Borneo), but individual cases have been increasingly identified in countries in Southeast Asia (*6*). Our large-scale molecular-based survey of malaria in Thailand during 2006–2007 showed that *P. knowlesi* was widely distributed at a low prevalence (in 0.57% of all malaria cases identified) in several malaria-endemic areas bordering Myanmar, Cambodia, and Malaysia (*7*). Correct diagnosis of malaria has a major effect on malaria control in terms of treatment outcomes, disease transmission, and interpretation of efficiency of a given control measure.

Although malaria caused by *P. knowlesi* is generally benign and responsive to chloroquine treatment, severe and fatal cases similar to complicated *P. falciparum* malaria cases have been documented (*6**,**8*). To date, it has been unknown whether human infections with *P. knowlesi* in Thailand were caused by a new emergence of this parasite species or whether the parasite had been circulating cryptically with other human malaria parasites. Furthermore, it would be useful to explore spatiotemporal distribution of malaria species in humans and analyze genetic characteristics of *P. knowlesi* circulating among naturally infected macaques and humans. These data could lead to a better understanding of malaria transmission and provide information for a more effective malaria control policy at a nationwide level. Therefore, we sought to determine the prevalence of this simian malaria in malaria-endemic regions of Thailand.

## Materials and Methods

### Prospective Study and Sample Collection

**Figure 1:**
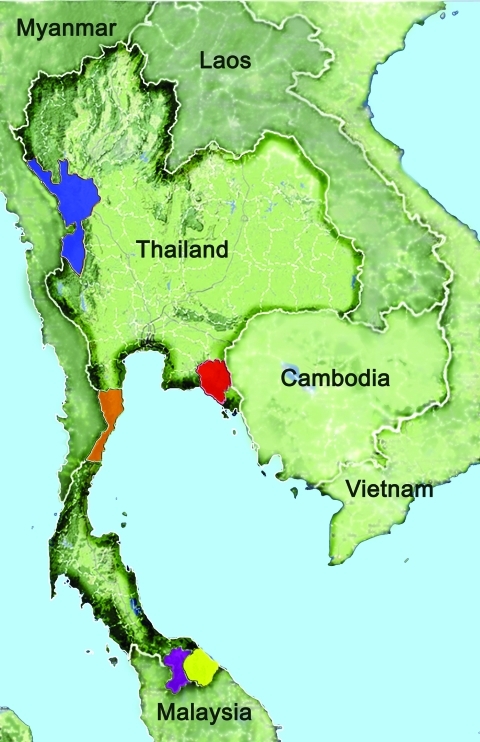
Provinces of Thailand where blood samples were obtained and tested for malaria, 1996–2009. Tak: blue, n = 210 in 1996, n = 681 in 2006–2007, and n = 1,216 in 2008–2009; Prachuab Khirikhan: orange, n = 215 in 2006–2007; Yala: purple, n = 286 in 2006–2007 and n = 1,408 in 2008–2009; Narathiwat: yellow, n = 370 in 2006–2007 and n = 421 in 2008–2009; and Chantaburi: red, n = 261 in 2006–2007 and n = 401 in 2008–2009.

Most malaria infections in Thailand occur in forests or forest fringes along its borders with other countries, and malaria transmission exhibits a bimodal pattern that peaks in May–July and October–November (*9**,**10*). During October 2008–September 2009, venous or finger prick blood samples were obtained from 3,770 febrile persons (2,577 male and 1,193 female; mean age 27.4 years, range 1–87 years) who came to malaria clinics in northwestern (Tak Province, n = 1,354), eastern (Chantaburi Province, n = 401), and southern (Yala Province, n = 1,552, and Narathiwat Province, n = 463) Thailand (Figure 1). These 3,770 persons represented 12.4% of the 30,425 malaria cases in these areas during the study period (*10*). A total of 470 blood samples from these persons were negative for malaria parasites by microscopy (153 in Tak, 179 in Yala, and 138 in Narathiwat). The study was reviewed and approved by the Institutional Review Board of Faculty of Medicine, Chulalongkorn University.

### Retrospective Study

A total of 210 blood samples obtained in 1996 from microscopy-positive symptomatic malaria patients (139 male and 71 female; mean age 25.1 years, range 12–72 years) living in Tak Province were included for comparative analysis. Of these samples, 143 were obtained during May–July; the remaining 67 samples were obtained during October–December. Blood samples were preserved in EDTA and stored at −40°C until use. Results of malaria species distribution during 2006–2007 from our analysis (*7*) were included for comparison.

### Microscopic Diagnosis

Thin and thick smears were prepared from each blood sample, stained with Giemsa solution, and examined by experienced microscopists for >200 leukocytes for a thick film and >200 microscopic fields with a 100× objective. Microscopists were blinded to clinical information and results of PCR detection.

### PCR-based Diagnosis

DNA was extracted from 200 μL of blood by using a DNA Minikit (QIAGEN, Hilden, Germany) according to the manufacturer’s instructions. Malaria species was identified by using a nested PCR with *Plasmodium* genus–specific outer primers (M18SF0: 5′-CCATTAATCAAGAACGAAAGTTAAGG-3′ and M18SR0c: 5′-TGTTGTTGCCTTAAACTTCCTTG-3′) derived from small subunit rRNA for a primary PCR (*7*). Nested PCR was conducted in separate tubes for each pair of species-specific primers: *P. falciparum*, PF18SF: 5′-CATCTTTCGAGGTGACTTTTAG-3′ and PF18SRc: 5′-GAAGAGAGAAATAGAGTAAAAAAC-3′; *P. vivax*, PV18SF: 5′-GAATTTTCTCTTCGGAGTTTATTC-3′ and PV18SRc: 5′-GTAGAAAAGGGAAAGGGAAACTGTTA-3′; *P. malariae*, PM18SF: 5′-GAGACATTCATATATATGAGTG-3′ and PM18SRc: 5′-GGGAAAAGAACGTTTTTATTAAAAAAAAC-3′; *P. ovale*, PO18SF: 5′-AATTCCTTTCGGGGAAATTTCTTA-3′ and PO18SRc: 5′-GGGAAAAGGACACATTAATTGTAT-3′; and *P. knowlesi*, PK18SF: 5′-GAGTTTTTCTTTTCTCTCCGGAG-3′ and PK18SRc: 5′-GGGAAAGGAATCACATTTAACGT-3′. Thermal cycling profiles for primary and nested PCRs contained 35 and 25 cycles (94°C for 40 s, 60°C for 30 s, and 72°C for 1 min), respectively. PCR products were analyzed by 2% agarose gel electrophoresis.

### Sequencing the Complete Merozoite Surface Protein 1 Gene of *P. knowlesi*

During December 2008–June 2009, a prospective survey of malaria in monkeys inhabiting Yala Province (n = 70) and Narathiwat Province (n = 566) showed that 5 *M. nemestrina* monkeys, 1 *M. fascicularis* monkey, and 1 *Semnopithecus obscurus* monkey in Narathiwat Province had *P. knowlesi* infections (*11*). The complete nucleotide sequences of the merozoite surface protein–1 gene of *P. knowlesi* (*Pkmsp-1*) from these pig-tailed macaques and humans in the current survey were obtained by direct sequencing of PCR-amplified products as described (*12*). Sequences have been deposited in GenBank under accession nos. JF837339–JF837353.

### Data Analysis

Sequences were aligned by using ClustalX with minor manual adjustments made by visual inspection (*13*). Phylogenetic construction was inferred from maximum-likelihood methods by using the Hasegawa, Kishino, and Yano model with 1,000 bootstrap iterations as implemented in MEGA version 5.01 (*14*). Differences between numbers of malaria cases were computed by using the Mann-Whitney U test, χ^2^ test, or Fisher exact test. A 2-tailed p value <0.05 indicated statistical significance.

## Results

### Malaria Species Distribution

During October 2008–September 2009, microscopic examinations of blood samples from 3,770 febrile patients showed that 3,300 had malaria parasites and 470 did not. Most malaria cases diagnosed by microscopy were either *P. falciparum* (51.55%) or *P. vivax* (48.21%); the prevalence of co-infections with both species was 0.18%. Of 432 samples containing co-infections with *P. falciparum* and *P. vivax* detected by PCR, only 3 (0.69%) samples were correctly diagnosed by microscopy. Microscopy showed negative results for 37 (8.56%) samples and failed to detect cryptic *P. falciparum* for 181 (41.90%) samples and cryptic *P. vivax* for 211 (48.84%) samples (Table 1). Conversely, 186 of 470 microscopy-negative samples were positive by nested PCR (79 *P. falciparum*, 68 *P. vivax*, 37 *P. falciparum* and *P. vivax*, 1 co-infection with *P. falciparum* and *P. malariae*, and 1 *P.* *knowlesi*); PCR failed to detect malaria in 46 microscopy-positive samples.

**Table 1:** Distribution of *Plasmodium* species in 3,770 febrile patients, Thailand, October 2008–September 2009

Species detected by PCR	Microscopy-based detection						PCR detection
*P. falciparum*	*P. vivax*	*P. malariae*	*P. ovale*	*P. falciparum* and *P. vivax*	Negative
*P. falciparum*	1,397	78	0	0	2	79	1,556
*P. vivax*	52	1,301	0	0	1	68	1,422
*P .malariae*	1	1	1	0	0	0	3
*P .ovale*	0	0	0	0	0	0	0
*P. knowlesi*	3	3	1	0	0	1	8
*P. falciparum* and *P. vivax*	211	181	0	0	3	37	432
*P. falciparum* and *P. malariae*	1	0	0	0	0	1	2
*P. falciparum* and *P. ovale*	3	0	0	0	0	0	3
*P. falciparum* and *P. knowlesi*	6	0	0	0	0	0	6
*P. vivax* and *P. malariae*	0	3	0	0	0	0	3
*P. vivax* and *P. ovale*	1	1	0	0	0	0	2
*P. vivax* and *P. knowlesi*	3	1	0	0	0	0	4
*P. falciparum, P. vivax,* and *P. knowlesi*	2	3	0	0	0	0	5
Negative	21	19	0	0	0	284	324
Total	1,701	1,591	2	0	6	470	3,770

A total of 3,446 blood samples contained malaria parasite DNA and showed greater differences in species distribution and mixed species infections than did microscopy (Table 1). Mixed species infections accounted for 13.26% of all PCR-positive cases and were most common in Tak Province (Table 2). Microscopy failed to detect *P. knowlesi* in these samples. In contrast, PCR identified 23 (0.67%) patients with *P. knowlesi* infections (8 monoinfections and 15 co-infections with other *Plasmodium* species) (Table 1).

**Table 2:** Distribution of *Plasmodium* species by malaria-endemic areas of Thailand, October 2008–September 2009*

Infection	Region, no. (%)				Total no. (%)
	Northwestern (Tak)	Eastern (Chantaburi)	Southern (Yala)	Southern (Narathiwat)	
Monoinfection	989 (81.33)	351 (87.53)	1,297 (92.12)	352 (83.61)	2,989 (86.74)
* P. falciparum*	507 (41.69)	27 (6.73)	810 (57.53)	212 (50.36)	1,556 (45.15)
* P. vivax*	481 (39.56)	320 (79.80)	485 (34.45)	136 (32.30)	1,422 (41.27)
* P. malariae*	1 (0.08)	1 (0.25)	0	1 (0.24)	3 (0.09)
* P. ovale*	0	0	0	0	0
* P. knowlesi*	0	3 (0.75)	2 (0.14)	3 (0.71)	8 (0.23)
Co-infection	227 (18.67)	50 (12.47)	111 (7.88)	69 (16.39)	457 (13.26)
*P. falciparum* and *P. vivax*	217 (17.85)	46 (11.47)	105 (7.46)	64 (15.20)	432 (12.54)
*P. falciparum* and *P. malariae*	1 (0.08)	0	0	1 (0.24)	2 (0.06)
*P. falciparum* and *P. ovale*	2 (0.16)	0	1 (0.07)	0	3 (0.09)
*P. falciparum* and *P. knowlesi*	2 (0.16)	0	3 (0.21)	1 (0.24)	6 (0.17)
*P. vivax* and *P. malariae*	1 (0.08)	0	2 (0.14)	0	3 (0.09)
*P. vivax* and *P. ovale*	1 (0.08)	0	0	1 (0.24)	2 (0.06)
*P. vivax* and *P. knowlesi*	2 (0.16)	1 (0.25)	0	1 (0.24)	4 (0.12)
*P. falciparum*, *P. vivax*, and *P. knowlesi*	1 (0.08)	3 (0.75)	0	1 (0.24)	5 (0.15)
Total	1,216	401	1,408	421	3,446

*Data from nested PCR. Percentages are of the total number in that region or infection category.

### *P. knowlesi* in Blood Samples Obtained in 1996

PCR analysis of 210 blood samples from microscopy-positive patients in Tak Province obtained during 1996 identified 55 and 96 patients as having monoinfections with *P. falciparum* (26.19%) and *P. vivax* (45.71%), respectively. The remaining 59 patients (28.10%) had mixed species infections with *P. falciparum* and *P. vivax* (n = 50), *P. vivax* and *P. malariae* (n = 7), *P. vivax* and *P. knowlesi* (n = 1), and *P. falciparum*, *P. vivax*, and *P. malariae* (n = 1). These findings indicate that *P. knowlesi* has circulated among humans in Thailand for at least 12–13 years.

### Spatial Variation

The distribution of *P. falciparum* and *P. vivax* displayed spatial variation in Thailand. PCR assays showed that *P. falciparum* and *P. vivax* contributed almost equally to malaria cases in Tak Province during 2008–2009 (a total of 50.55% and 48.68%, respectively), *P. vivax* was more prevalent than *P. falciparum* in Chantaburi Province, and *P. falciparum* was more prevalent than *P. vivax* in Yala and Narathiwat Provinces. Despite a low overall prevalence of the remaining malaria species (<2%), *P. knowlesi* could be detected more often than *P. malariae* and *P. ovale* in these malaria-endemic areas (Table 3).

**Table 3:** Temporal variation in distribution of *Plasmodium* species by malaria-endemic region of Thailand, 2006–2009*

Species	% Distribution										
	Northwestern (Tak)			Eastern (Chantaburi)			Southern (Yala)			Southern (Narathiwat)	
	2006–2007, n = 681	2008–2009, n = 1,216		2006–2007, n = 261	2008–2009, n = 401		2006–2007, n = 286	2008–2009, n = 1,408		2006–2007, n = 370	2008–2009, n = 421
*P. falciparum*	44.54	50.55		8.42	16.74		57.14	60.50		23.31	56.82
*P. vivax*	52.63	48.68		91.03	81.50		41.86	38.97		76.17	41.34
*P. malariae*	1.16	0.21		0	0.22		0	0.13		0	0.41
*P. ovale*	1.41	0.21		0.27	0		0	0.07		0	0.20
*P. knowlesi*	0.26	0.35		0.27	1.54		1.00	0.33		0.52	1.22

*Data from nested PCR. Temporal variation in distribution in 1996 (n = 210) was 39.26 for *Plasmodium falciparum*, 57.41 for *P. vivax*, 2.96 for *P. malariae*, 0 for *P. ovale*, and 0.37 for *P. knowlesi*.

### Temporal Variation

Distribution of *P. falciparum*, *P. vivax*, *P. malariae*, and *P. ovale* in Thailand among samples obtained during 2006–2007 and 2008–2009 exhibited significant temporal variation (χ^2^ 99.9, 92.2, 24.6, and 20.5, respectively; all p values <0.0001). Conversely, distribution of *P. knowlesi* remained stable over these periods (χ^2^ 0.17, p = 0.68). If one considers that samples from 3 periods (1999, 2006–2007, and 2008–2009) were available for comparison only for Tak Province, the contribution of *P. knowlesi* to malaria cases was not different in all pairwise comparisons, which suggested stable prevalence of this simian malaria for more than a decade in this malaria-endemic area. In contrast, a significant difference was found in the distribution of *P. falciparum*, *P. vivax*, and *P. malariae* obtained during the 3 periods in each malaria-endemic area (p<0.05 for all pairwise comparisons), and *P. ovale* was not detected in samples obtained during 1996 but comprised 1.41% and 0.21% of samples obtained during 2006–2007 and 2008–2009, respectively (Table 3).

Prevalence of mixed species infections showed regional and seasonal variations characterized by a significantly higher prevalence during the dry season than in the rainy season in isolates from Chantaburi Province (χ^2^ 5.94, p = 0.015; odds ratio [OR] 2.59, 95% confidence interval [CI] 1.20–5.60). The opposite result was observed for isolates from Yala Province (χ^2^ 101.7, p<0.001; OR 7.2, 95% CI 4.65–10.90). No significant seasonal difference in prevalence of mixed species infections occurred in isolates from Tak Province (χ^2^ 1.09, p = 0.60; OR 1.09, 95% CI 0.81–1.46) and Narathiwat Province (χ^2^ 0.10, p = 0.76; OR 1.09, 95% CI 0.64–1.87).

### Characteristics of *P. knowlesi*–infected Patients

Of 5,254 PCR-positive samples obtained during 1996, 2006–2007, and 2008–2009, *P. knowlesi* was detected in 34 patients (0.65%) (Table 2, Table 3) (*7*). All patients infected with *P. knowlesi*, including the first case-patient identified in 2000 (n = 35), had uncomplicated malaria symptoms (*3**,**7*). Age range of *P. knowlesi*–infected patients was 4–59 years (mean 30 years, mode 19 years, median 33.5 years), and most (73.50%) cases occurred in persons 16–45 years of age. *P. knowlesi* malaria was diagnosed in male patients ≈2× more often than in female patients (M:F ratio 2.18:1), which was similar to the sex distribution of total malaria cases (M:F ratio 2.16:1). Thus, no sex preference was observed for *P. knowlesi* (p = 0.97).

Almost half of the *P. knowlesi*–infected malaria patients acquired infections in southern Thailand near Malaysia. Monoinfections with *P. knowlesi* were observed in 10 patients whose parasite densities ranged from 0 to 145,000 parasites/μL. The remaining 24 patients had co-infections with *P. falciparum* (n = 16) or *P. vivax* (n = 9) or triple infections with *P. falciparum* and *P. vivax* (n = 5). Although the geometric mean parasite density for *P. knowlesi* moninfections was higher than that for mixed species infections, no significant difference was observed (Table 4).

**Table 4:** Parasite densities of patients with *Plasmodium knowlesi* monoinfection and co-infection with other malaria species, Thailand, October 2008–September 2009*

Category	Parasite density, parasites/μL†		
	Geometric mean	Ratio of geometric means‡	Range
*P. knowlesi* monoinfection, n = 10	4,165	NA	0–145,000
*P. knowlesi* co-infection with			
*P. falciparum*, n = 11	1,632	2.55	440–6,560
*P. vivax*, n = 9	1,686	2.47	320–13,120
*P. falciparum* and *P. vivax*, n = 5	487	8.55	320–1,520
All co-infections, n = 25	1,271	3.28	320–13,120

*NA, not applicable. †Parasite densities between categories were not significantly different (p>0.05, by Mann-Whitney *U* test). ‡Ratio of parasite densities of monoinfection to co-infection.

Initial microscopic diagnosis identified *P. malariae* for patients with moninfections and *P. falciparum* or *P. vivax* for patients with mixed species infections. Approximately two thirds (23/35) of *P. knowlesi* malaria occurred in the rainy season. However, the ratio of *P. knowlesi*–infected patients to total malaria patients for the rainy and dry seasons was not significantly different (χ^2^ 0.25, p = 0.62; OR 1.20, 95% CI 0.60–2.38). Most (73.50%) human infections with *P. knowlesi* occurred in areas with macaques living nearby.

### Msp-1 Gene Sequence of *P. knowlesi*

Fifteen complete *Pkmsp-1* gene sequences were analyzed for 10 isolates from humans and 5 isolates from pig-tailed macaques. These human isolates were obtained from 8 patients in the current survey (3 from Narathiwat Province, 3 from Chantaburi Province, and 2 from Yala Province) and from 2 patients from Prachuab Khirikhan Province obtained during 2006–2007 (*7*). The monkey isolates were obtained from 5 naturally infected pig-tailed macaques from Narathiwat Province during the same period as the current malaria survey in humans (*11*). Each isolate had single *Pkmsp-1* gene sequences because no superimposed results were observed in electropherograms. Size variation (range 5,430–5,613 bp) was observed among *Pkmsp-1* genes of these isolates.

**Figure 2:**
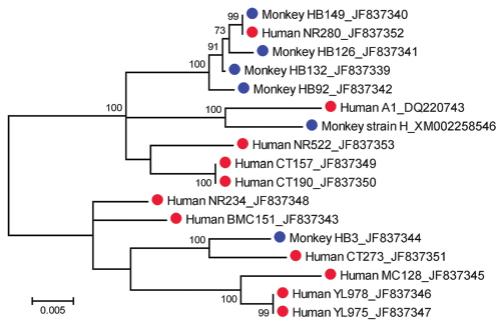
Maximum-likelihood tree inferred from the complete merozoite surface protein 1 gene sequences of *Plasmodium knowlesi* from humans (red circles) and macaques (blue circles). The tree is drawn to scale, and branch lengths are measured in number of substitutions per site by using MEGA version 5.01 (*14*). Bootstrap values >50% from 1,000 iterations are shown. Human isolates are from the following provinces: Narathiwat (NR280, NR234, and NR522); Yala (YL975 and YL978); Chantaburi (CT157, CT190, and CT273); and Prachuab Khirikhan (BMC151, MC128, and DQ220743). Isolates HB3, HB92, HB126, HB132, and HB149 are from macaques in Narathiwat Province. GenBank accession nos. are shown after isolate names.

Sequence analysis identified conserved and variable domains in *Pkmsp-1* genes, similar to those found in the *msp-1* gene of *P. vivax* (*15*). Nucleotide substitutions in conserved regions showed a dimorphic pattern. Phylogenetic analysis showed that human and macaque isolates had genetic diversity in the *Pkmsp-1* gene that could be located in 2 clusters with 100% bootstrap support, which confirmed dimorphism of this gene. One cluster contained 6 human isolates (BMC151, CT273, MC128, NR234, YL975, and YL978) and a monkey isolate from Narathiwat Province (isolate HB3), and the remaining isolates belonged to the other cluster (Figure 2). The *Pkmsp-1* gene sequence from 1 patient (NR280) in Narathiwat Province was identical with that isolated from a pig-tailed macaque (HB149) living in the same locality. Furthermore, isolates from 2 patients (YL975 and YL978) who lived in Yala Province had identical *Pkmsp-1* gene sequences. The patients were concurrently infected with *P. knowlesi* in the same malaria-endemic areas. The other identical sequences were found in isolates from 2 patients (CT157 and CT190) in Chantaburi Province who had onset of febrile illness a few days apart (Figure 2).

## Discussion

Our PCR analysis identified spatiotemporal heterogeneity in the prevalence of *P. falciparum* and *P. vivax*, which is consistent with that of previous analyses by microscopy-based detection (*16**,**17*). Prevalence of *P. falciparum* and *P. vivax* in adjacent Yala and Narathiwat Provinces also showed a spatial difference in species distribution during 2006–2007 but no difference during 2008–2009. These results supported a spatiotemporal pattern on a microgeographic level as reported for other malaria-endemic areas in Thailand (*16*). Furthermore, the prevalence of *P. malariae* and *P. ovale* varied according to location and time.

Cross-border migration of infected persons, predominantly along the Thailand–Myanmar and Thailand–Cambodia borders, could contribute spatial and temporal variation in the prevalence of these malaria parasite species (*16**,**17*). However, migration per se could not explain similar findings in Yala and Narathiwat Provinces because almost all malaria cases in these areas were autochthonous (*10*). A dramatic increase in *P. vivax* prevalence in malaria-endemic areas bordering Cambodia during the past decade has reportedly been caused by relative changes in species distribution of local vectors and differences in vector capacity (*18**–**20*). Furthermore, intrinsic differences in parasite biology such as hypnozoites in *P. vivax* and *P. ovale* could contribute to additional episodes of infections if these infections are not radically treated. Therefore, spatiotemporal variation in these human cases of malaria in Thailand likely results from multiple variable factors.

Our previous survey in 2006–2007 showed that co-infections with different malaria parasite species displayed spatial variation, characterized by a high prevalence (≈23%–24%) along the Thailand–Myanmar border that contrasts with a low prevalence of 3% and 5% in malaria-endemic areas bordering Cambodia and Malaysia, respectively (*7*). Our current survey has detected different prevalences of mixed species infections than during 2006–2007 in these same malaria-endemic areas that could have been caused by different prevalences of each malaria parasite species.

Microscopy usually did not detect cryptic *P. falciparum* or cryptic *P. vivax* in samples containing both species identified by PCR (41.90% and 48.84%, respectively). When 1 of these 2 species coexisted with other malaria species (including *P. knowlesi*), only *P. falciparum* or *P. vivax* infection was diagnosed by microscopy. Failure to diagnose mixed species infections could result in repeated diagnosis and treatment, economic losses, misinterpretation of drug or vaccine efficacies, and inadequate control policies (*21*).

Co-infections with *P. falciparum* and *P. vivax* could be advantageous to hosts in terms of reduced disease severity (*22*), less chance for gametocyte carriage (*23*), and decreased parasite density (*24*). However, detrimental outcomes have been observed in other studies (*25**–**27*). Although parasite densities of *P. knowlesi* monoinfections and co-infections with other species were not different, the geometric mean of monoinfections was 2× that of co-infections (Table 4). Therefore, interference by a high prevalence of mixed species infections (≈14% for the current survey) and cross-species immunity may contribute to the low prevalence of *P. knowlesi* in Thailand. In contrast, mixed species infections were less prevalent in Sarawak, where most (≈91%) malaria patients had *P. knowlesi* monoinfections (*4**,**6*).

No difference in temporal and spatial distribution of *P. knowlesi* was found in the study areas, which suggested that transmission patterns could be different from those for human malarias caused by other parasites. The natural vectors for *P. knowlesi* in the Malay Peninsula are *Anopheles cracens* and *An. latens* mosquitoes, which are members of the Leucosphyrus group (*28**,**29*). The main vectors for human malaria in Thailand are *An. minimus*, *An. maculates*, and *An. dirus* mosquitoes (*30*). Although *An. dirus* mosquitoes also belong to the Leucosphyrus group and have been identified as potential vectors for *P. knowlesi* in Vietnam (*31*), this vector species has drastically decreased in abundance in all major malaria-endemic areas of Thailand during the past decade (C. Putaporntip et al., unpub. data), although feeding patterns of these mosquitoes are zoonotic rather than anthropophilic in certain areas (*32*). Therefore, transmission of *P. knowlesi* to humans could be limited and distinct from transmission by other species that cause malaria in humans in Thailand. Conversely, identification of *P. knowlesi* cryptically circulating among malaria patients in Tak Province obtained during 1996 has implied its occurrence at least 12–13 years ago in Thailand and a relatively stable prevalence. Likewise, recent analysis of archive blood samples for patients in Sarawak obtained more than a decade ago has supported the suggestion that *P. knowlesi* is not a newly emergent zoonotic malaria species in humans (*33*).

Our recent survey of simian malaria in long-tailed and pig-tailed macaques in Thailand (n = 754) showed that *P. knowlesi* had a prevalence of 5.6% and 2.3%, respectively (*11*). Sequence analysis of *Pkmsp-1* genes from 5 *P. knowlesi*–infected macaques living near infected humans has shown that all parasites had unique sequences, which reaffirmed genetic heterogeneity of *P. knowlesi* in its natural hosts in Thailand (*7*). *P. knowlesi* isolated from a patient in Narathiwat Province shared an identical *Pkmsp-1* gene sequence with that from a pig-tailed macaque living nearby, which suggested that *P. knowlesi* could be transmitted from macaques to humans and vice versa through anopheline vectors. However, the higher prevalence of *P. knowlesi* in macaques than in humans and the chronic course of parasitemia in asymptomatic macaques could increase the likelihood of transmission from macaques to humans. Furthermore, the acute clinical course of malaria infection in humans would be rapidly eliminated by antimalarial treatment and result in a lower likelihood of additional transmission of *P. knowesi* gametocytes to vectors (*1**,**7*).

Identical *Pkmsp-1* gene sequences found in 2 patients in Chantaburi Province who lived in the same area and had symptomatic malaria during the same week suggests that both patients acquired the infections from a common source. Alternatively, parasites harboring this identical *Pkmsp-1* allele could predominate among infected macaques in the region. Unfortunately, no extensive survey of malaria in wild macaques in Chantaburi Province was performed during the study. Despite limited number of samples in our study, *Pkmsp-1* gene sequences from humans in eastern and southern Thailand were found in both clusters of the phylogenetic tree, which suggested that both dimorphic types are widely distributed in Thailand. Whether both dimorphic types of *Pkmsp-1* genes from infected persons co-exist in other malaria-endemic regions of Southeast Asia remains to be investigated.

A spectrum of clinical manifestations has been observed in persons infected with *P. knowlesi* (*6**,**34*). However, most patients with *P. knowlesi* malaria had febrile illness and associated symptoms that were indistinguishable from those caused by other malaria species (*2**,**3*). *P. knowlesi* requires 24 hours to complete its asexual erythrocytic cycle, which results in a unique quotidian type of fever pattern different from that for the other 4 human malaria species (*1*). However, such paroxysms have no practical value for presumptive diagnosis because characteristic fever patterns would not be observed during early phase of infections, and mixed species infections could further complicate febrile symptoms (*35*). Furthermore, *P. knowlesi* malaria is responsive to chloroquine treatment and would likely be responsive to other antimalarial drugs (*4*). Therefore, reappearance of *P. knowlesi* among patients with mixed infections after initial treatment for infection with another malaria species in Thailand seems less likely. *P. knowlesi* infections in humans caused severe symptoms and deaths in 6.5% and 1.8%, respectively, in the Kapit population in Sarawak where it comprised 70% of all malaria cases (*4*). However, if *P. knowlesi* malaria had a high prevalence of complicated symptoms, the probability of observing such severe and fatal consequences from *P. knowlesi* infections among patients in Thailand would be low.

In conclusion, human malaria caused by *P. knowlesi* has occurred in Thailand for more than a decade. Despite variations in the prevalence of all 4 human malaria species, *P. knowlesi* has shown stable prevalence rates, which suggests different transmission cycles. Human infections with *P. knowlesi* in Thailand could result from macaques, as shown by identical *Pkmsp-1* gene sequences of human and macaque origins.
